# Neutrophil Activation in Acute Hemorrhagic Fever With Renal Syndrome Is Mediated by Hantavirus-Infected Microvascular Endothelial Cells

**DOI:** 10.3389/fimmu.2018.02098

**Published:** 2018-09-18

**Authors:** Tomas Strandin, Satu Mäkelä, Jukka Mustonen, Antti Vaheri

**Affiliations:** ^1^Department of Virology, Medicum, Faculty of Medicine, University of Helsinki, Helsinki, Finland; ^2^Department of Internal Medicine, Faculty of Medicine and Life Sciences, Tampere University Hospital, University of Tampere, Tampere, Finland

**Keywords:** hantavirus, HFRS, neutrophils, IL-8, endothelial cells, degranulation, NETs, NETosis

## Abstract

Hantaviruses cause hemorrhagic fever with renal syndrome (HFRS) and hantavirus cardiopulmonary syndrome (HCPS) in humans. Both diseases are considered to be immunologically mediated but the exact pathological mechanisms are still poorly understood. Neutrophils are considered the first line of defense against invading microbes but little is still known of their role in virus infections. We wanted to study the role of neutrophils in HFRS using blood and tissue samples obtained from Puumala hantavirus (PUUV)-infected patients. We found that neutrophil activation products myeloperoxidase and neutrophil elastase, together with interleukin-8 (the major neutrophil chemotactic factor in humans), are strongly elevated in blood of acute PUUV-HFRS and positively correlate with kidney dysfunction, the hallmark clinical finding of HFRS. These markers localized mainly in the tubulointerstitial space in the kidneys of PUUV-HFRS patients suggesting neutrophil activation to be a likely component of the general immune response toward hantaviruses. We also observed increased levels of circulating extracellular histones at the acute stage of the disease supporting previous findings of neutrophil extracellular trap formation in PUUV-HFRS. Mechanistically, we did not find evidence for direct PUUV-mediated activation of neutrophils but instead primary blood microvascular endothelial cells acquired a pro-inflammatory phenotype and promoted neutrophil degranulation in response to PUUV infection *in vitro*. These results suggest that neutrophils are activated by hantavirus-infected endothelial cells and may contribute to the kidney pathology which determines the severity of HFRS.

## Introduction

Hantaviruses are the causative agents of two human diseases: hemorrhagic fever with renal syndrome (HFRS) in Eurasia and hantavirus cardiopulmonary syndrome (HCPS) in the Americas. Each hantavirus species is carried by its specific rodent host with no or minimal signs of disease but cause occasional human spillover infections with immune-mediated pathology (1). A hallmark of both hantavirus diseases is increased vascular permeability which mediates kidney and lung failure associated with HFRS and HCPS, respectively (1). Endothelial cells lining the vasculature are also the prime target of viral replication in patients (2, 3). Puumala hantavirus (PUUV) circulates in Northern Europe and Russia causing a relatively mild form of HFRS as compared to Hantaan (HTNV) or Dobrava hantavirus (DOBV)-caused HFRS and especially Andes (ANDV)- or Sin Nombre (SNV)-caused HCPS in which fatality rates can reach 40% (4).

Typical laboratory findings in acute PUUV-caused HFRS are leukocytosis, thrombocytopenia, increased C-reactive protein (CRP) level, and as signs of acute kidney injury (AKI), proteinuria, haematuria and elevated serum creatinine concentration (5). In addition to thrombocytopenia, hematological abnormalities include increased coagulation and fibrinolysis, complement activation, and elevated levels of pro-inflammatory cytokines which all have the potential to contribute to vascular permeability. Vascular permeability, in turn, could be the underlying cause of proteinuria which typically precedes AKI (6). The pathophysiology of PUUV-HFRS associated AKI is usually described as tubulointerstitial nephritis and infiltration of several immune cell types such as lymphocytes, monocytes, and polymorphonuclear leukocytes into kidneys have been observed (7, 8).

Neutrophils are the most abundant circulating leukocytes in humans and play a fundamental role in the innate immune response (9). Circulating neutrophils are the first type of immune cells recruited to sites of inflammation or infection. In humans, one of the most important chemotactic factors for neutrophils is interleukin (IL)-8, released at the site inflammation/infection (10). After receiving chemotactic signals neutrophils interact with endothelial cells lining the vasculature in order to traverse the endothelium and into the inflamed tissues (11). *In vivo*, the interactions between neutrophils and endothelial cells include initial rolling followed by firm adhesion and finally transendothelial migration. Firm adhesion is facilitated, among other factors, by endothelial intercellular adhesion molecule (ICAM)-1 expressed on the surface of endothelial cells and CD11b/CD18 integrin complex (also known as Mac-1, CR3 or α_M_β_2_) on neutrophils (12). Once neutrophils reach the site of infection their primary role is to kill invading microbes by production of reactive oxygen species (ROS) and the release of antimicrobial proteins such as myeloperoxidase (MPO) and human neutrophil elastase (HNE) in a process of degranulation (13, 14). In addition to ROS formation and degranulation, neutrophils are also able to release neutrophil extracellular traps (NETs), consisting of extracellular chromatin decorated with histones and granular proteins such as MPO and HNE, and with the potential to entrap and kill pathogens (15, 16).

The activation of neutrophils in several bacterial infections is well-described but their role in virus-mediated diseases has been neglected to a large extent (17). The involvement of neutrophils in the pathogenesis of hantavirus diseases is suggested by acutely increased serum levels of cytokines and chemokines with known functions in neutrophil chemotaxis, differentiation and mobilization (18–20). Increased numbers of circulating histones, cell-fee DNA and histone-DNA complexes have been described in the acute stage of PUUV infection (21–23), suggesting that neutrophils release NETs during HFRS. NETosis could potentially explain capillary leakage in HFRS (24). Interestingly, neutrophils are crucial for increased vascular permeability observed in response to HTNV infection in SCID mice (25). Furthermore, HTNV has also been shown to cause NETosis *in vitro* (21).

In this study we wanted to elucidate the role of neutrophil activation in HFRS by determining markers of neutrophil activation (MPO, HNE, histones, and IL-8) in blood and tissues of patients suffering from acute PUUV-caused HFRS. In addition, to directly determine the role that hantavirus plays in mediating neutrophil responses in PUUV-HFRS, we investigated the potential of purified PUUV or PUUV-infected endothelial cells to activate neutrophils *in vitro*. Taken together, we found that the levels of circulating and tissue-localized MPO, HNE and IL-8 are elevated in acute PUUV-HFRS and correlate with kidney dysfunction, thereby corroborating the role of neutrophils in hantavirus pathogenesis. Mechanistically, our results do not support direct virus-mediated neutrophil activation but rather an indirect mechanism through infected endothelial cells. Finally, the antiviral function of neutrophils was pinpointed strongly to the release of proteases from neutrophils.

## Materials and methods

### Patient samples

The study was approved by the Ethics Committee of Tampere University Hospital (Nos. 99256 and R04180). All subjects gave written informed consent in accordance with the Declaration of Helsinki. The study consisted of plasma samples from patients treated for serologically confirmed acute PUUV infection at the Tampere University Hospital, Finland, during September 2000–March 2009. We assessed the extracellular circulating levels of MPO, HNE, histone H3 and IL-8 in plasma samples obtained from patients with acute PUUV-caused HFRS at 1st day of hospitalization (acute stage; median days after onset of fever 4 ± 2), early recovery phase (15–30 days after hospitalization) and healthy controls.

The study included Boiun-fixed, paraffin-embedded kidney biopsies obtained at Tampere University Hospital during 1985–1987. The biopsies among patients with PUUV-HFRS were performed for clinical reasons at the time when there was no reliable serological test for PUUV infection available. All these biopsies were performed during the acute phase of the disease. The highest measured serum creatinine level of the patients ranged from 220 to 1,050 μmol/L. The biopsy findings were acute interstitial in two cases and acute tubulointerstitial nephritis in three, both findings being typical for PUUV-induced AKI (5).

PUUV-negative cases served as controls. Indications for renal biopsies were AKI in one case, microscopic hematuria and/or proteinuria in four cases. The biopsy findings were normal morphology in two cases, acute tubulointerstitial nephritis, mesangial proliferative glomerulonephritis and IgM-glomerulonephritis in one case each. In both groups of biopsy patients a positive (PUUV-HFRS cases) or negative (controls) PUUV-serology was determined using stored serum samples obtained at the time of renal biopsy.

### Primary antibodies

Primary antibodies used in this study were rabbit polyclonal antibodies to MPO (Thermo scientific, #RB-373-A), Histone H3 (Abcam, #ab18521) or HNE (Abcam, #ab68672) and mouse monoclonal antibodies to IL-8 (RnD Systems; #Mab-208), CD18 (Millipore, #TS1-18) and ICAM-1 (RnD systems, #BBIG-I1) in addition to IgG1 isotype control (Immunotools). Rabbit polyclonal antibodies for PUUV nucleocapsid protein and glycoproteins have been described before (26).

### Histone quantification

Histones were measured by a dot-blot assay where 2 μl of patient or healthy control EDTA-plasma were pipetted on nitrocellulose membrane, air-dried and probed with Histone H3 antibody in blocking buffer [1.5% milk in Tris–EDTA–NaCl–Tween (TENT)]. After washing with TENT, the primary antibody was detected with IR800-conjugated anti-rabbit antibody (Li-cor) in blocking buffer. After additional washing, signal intensity was determined by Odyssey instrumentation (Li-cor). Recombinant Histone H3 (New England Biolabs) was used for standard preparation.

### Enzyme-linked immunosorbent assays (ELISAs)

The levels of MPO, HNE, and IL-8 were measured from patient or control plasma using ELISA kits provided by Abcam (for MPO, HNE, and IL-8) or Immunotools (for IL-8).

### Immunohistochemistry (IHC)

IHC was performed on kidney sections after heat-mediated antigen retrieval using the protocol provided in Vectastain ABC Elite HRP kit from Vector labs. Avidin/biotin blocking kit, biotinylated goat anti-mouse IgG or anti-rabbit secondary antibodies and DAB substrate were used as suggested by the vendor (Vector labs). The mean percentage of DAB positive area was counted from four images taken (1.5 × 1.5 mm) from each individual section using Fiji Image J software.

### Cultures of primary blood microvascular endothelial cells (BECs)

BECs were obtained from Lonza and maintained in endothelial basal medium (EBM-2) supplemented with SingleQuots™ Kit containing 5% fetal bovine serum (FBS), human endothelial growth factor, hydrocortisone, vascular endothelial growth factor, human fibroblast growth factor-basic, ascorbic acid, R^3^-insulin like growth factor-1, gentamicin and amphotericin-B (Lonza). For experiments the cells were used at passages 7–10.

### Virus propagation and titration

PUUV Kazan strain was propagated in Vero E6 cells (green monkey kidney epithelial cell line; ATCC no. CRL-1586) grown in Minimum Essential Medium containing 10% FCS, penicillin and streptomycin (cMEM). For experiments, PUUV was purified from Vero E6 cell supernatant through a 30% sucrose cushion by ultracentrifugation. Virus titers were measured by incubating diluted virus stocks on Vero E6 cells for 24 h at 37°C and subsequently staining acetone-fixed cells with a polyclonal antibody specific for PUUV nucleocapsid protein and AlexaFluor488-conjugated secondary antibody. Focus-forming units (FFU)/ml were counted under an UV-microscope.

### Infection of BECs

Confluent BECs were infected for 1 h at 37°C using purified PUUV diluted in BEC growth medium at multiplicity of infection (MOI) of 10. For inactivation of PUUV, virus stocks were kept under UV light for 30 min.

### Immunofluorescence

Immunofluorescence of either mock-, UV-PUUV or live PUUV-infected BECs was performed on cells grown on black, glass-bottomed cell culture plates. After fixation with 4% formaldehyde, cells were permeabilized (3% BSA, 0.3% TritonX-100) for 10 min and incubated with primary antibodies to MPO, HNE, ICAM-1 or viral nucleocapsid protein followed by appropriate Alexafluor488- or Alexafluor594-conjugated secondary antibodies (Thermo Scientific). To stain the nuclei, cells were incubated with Hoechst 33258. After washing, fluorescence intensity was counted using Hidex sense microplate reader (Hidex) and images taken using Leica TCS SP8 X confocal microscope (Biomedicum Imaging Unit, Biomedicum, University of Helsinki, Helsinki, Finland). Neutrophil activation was quantitated by the percentage of decondensed polymorphonuclear cells with simultaneous MPO relocalization in 4 immunofluorescence images (500 × 500 μm) taken randomly from each well by confocal microscope.

### PMN cultures

Fresh blood from healthy volunteers was drawn into EDTA-tubes and PMNs immediately isolated using Polymorphoprep separation medium (Axis-Shield) according to manufacturer's protocol. PMN purity and viability were assessed by phenotypic polymorphonuclear characterization using Hoechst 33258 fluorescence microscopy and trypan blue exclusion test, respectively (both were routinely found to be over 90%). Isolated PMNs were diluted into BEC growth medium (10^6^ cells/ml) and incubated with mock-, UV-PUUV or live PUUV-infected BECs for 1 or 3 h at 37°C (10 times excess PMNs over BECs). Cells were washed and subjected directly to immunofluorescence staining as described above for determination of PMN binding. When PMN-BEC co-cultures were incubated in the presence of 10 μg/ml neutralizing antibodies to IL-8, CD18 or viral glycoproteins, PMNs were pre-treated with FcR blocking reagent (Immunostep) for 10 min.

### Neutrophil activation assays

Purified virus or virus-containing Vero E6 cell culture supernatants were incubated with freshly isolated PMNs (MOI 1) in cMEM for 3 h at 37°C. The incubation time was chosen based on the optimal time needed to detect adequate levels of PMN activation marker expression with low concurrent spontaneous activation due to culturing. After fixation with 2% formaldehyde, extracellular DNA was quantitated with the fluorescent, cell-impermeable Pico-Green dsDNA binding reagent (Thermo Scientific) using Hidex Sense microplate reader. Peroxidase activity was assessed from supernatants of pelleted PMNs (400 × g 5 min) by the chromogenic peroxidase substrate 3,3′,5,5′-tetramethylbenzidine (TMB).

### PUUV viability assay

Purified PUUV was incubated for 3 h with freshly isolated PMNs (in a 1:1 ratio) in assay buffer (10 mM Hepes pH 7,4; 150 mM NaCl). Neutrophil activator phorbol myristate acetate (PMA; 1 μg/ml) and one of the following inhibitors NaN_3_ (0.01 or 0.002%), PMSF (0.2 or 1 mM), EDTA (2 mM) or DNAse I (10 U/ml) were added where indicated. After incubation, PMNs were pelleted by centrifugation (400 × g 5 min) and one third of the supernatant used for PUUV titer measurement as described above. The rest of the sample was used for immunoblotting by standard procedures for PUUV structural proteins (27) using polyclonal antibodies specific for Gn, Gc or N followed by IRD800-conjugated secondary antibody (Li-Cor) and detection by Odyssey.

### Statistics

Significant differences between groups of normally distributed data was assessed by student's *T*-test and non-normally distributed data by Mann-Whitney *U*-test or Kruskal-Wallis *H*-test for multiple populations. The normality of data was estimated by Shapiro-Wilk test. Correlations between parameters were assessed using Spearman's rank correlation test. All analyses were done with SPSS software version 24 (SPSS Inc., Chicago, IL, USA) or GraphPad prism (La Jolla, CA, United States).

## Results

### Circulating levels of MPO, HNE, histones, and IL-8 are elevated in acute PUUV-caused HFRS

**Figure 1:**
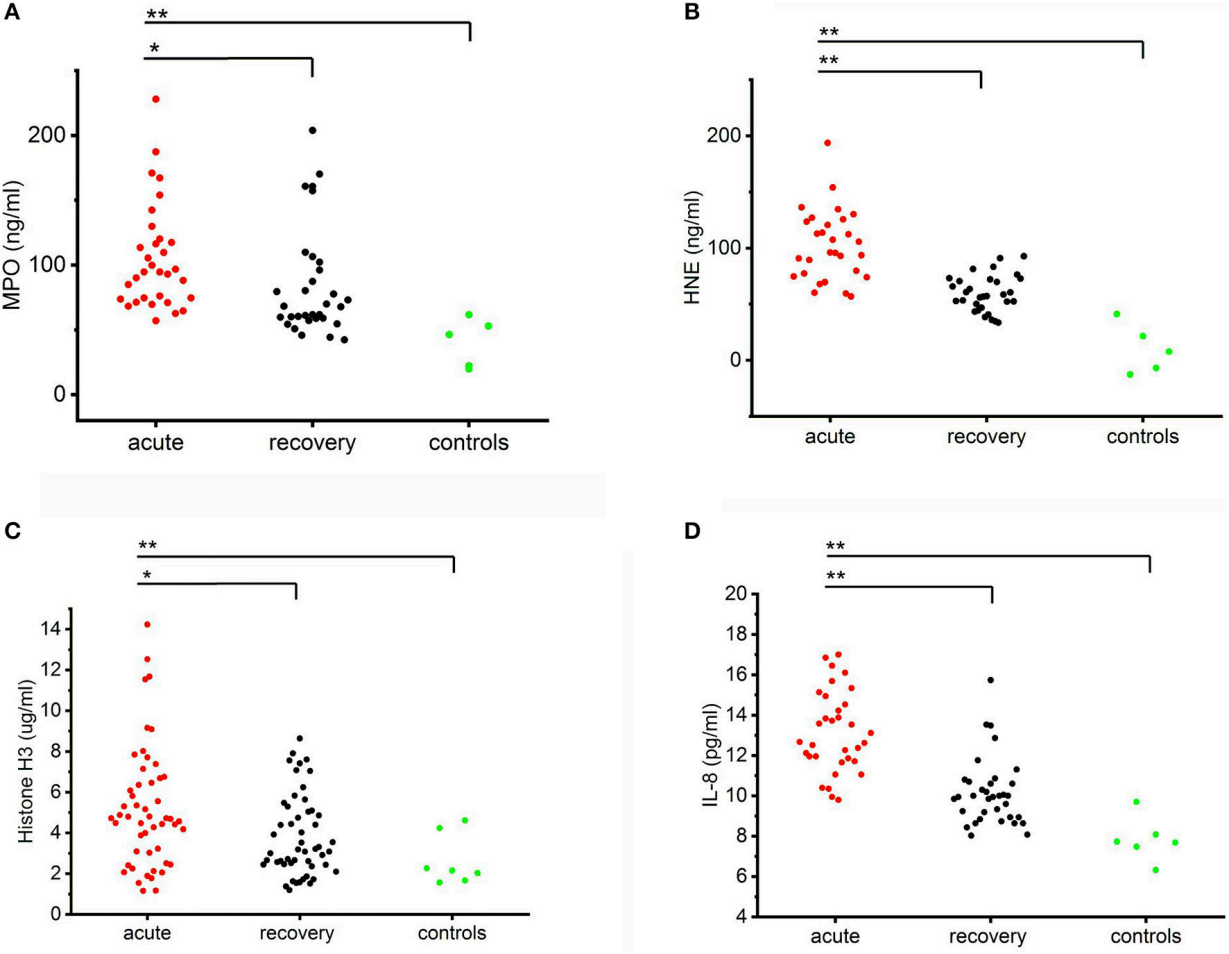
Circulating levels of MPO, HNE, histone H3 and IL-8 in PUUV-HFRS. Plots of the acute (median days after onset of fever 4 ± 2) and recovery stage (15–30 days post onset) levels of MPO **(A)**, HNE **(B)**, histones **(C)** or IL-8 **(D)** in PUUV-infected patient plasma samples (*n* = 32, 30, 53 and 36, respectively) as compared to healthy controls (*n* = 5, 5, 5, and 8, respectively) distributions across groups were compared by Kruskal-Wallis H test and statistically significant differences indicated as either ^*^p < 0.05 or ^**^p < 0.01.

Circulating levels of MPO, HNE, histones, and IL-8 were all significantly higher in the acute stage of the disease as compared to the recovery stage or healthy controls (Figure 1). The levels of all the measured markers remained elevated also in the recovery stage as compared to controls but this difference was not statistically significant. Strongly elevated circulating levels of MPO and HNE suggest that neutrophils are activated in acute PUUV-HFRS. Furthermore, increased numbers of extracellular histones imply that neutrophil activation could, at least in part, be due to NETosis (21). Interestingly, in the present study, neutrophil activation was accompanied by elevated levels of IL-8, a chemotactic and priming factor for neutrophils.

To get further insight to the role of neutrophil activation in the pathogenesis of PUUV-HFRS by we correlated the acute plasma levels of MPO, HNE, histone H3, and IL-8 to variables reflecting severity of AKI (maximum plasma creatinine level measured during the hospital stay), and to hematological variables (minimum blood platelet count, maximum blood leukocyte count, and plasma tissue plasminogen activator (tPA) level) (Table 1). We found that HNE and histone H3 positively correlated with the severity of AKI. In addition, MPO and HNE correlated with all the tested hematological variables. Not surprisingly, given the likely neutrophil origin of MPO and HNE, they also correlated strongly with each other. The acute IL-8 levels correlated significantly with low platelets and exceptionally strongly with MPO, HNE and histones (*p* < 0.005 for all) suggesting that IL-8 could play a role in neutrophil activation.

**Table 1:** Correlation between different clinical variables reflecting HFRS disease severity and markers of neutrophil activation.

		**Creatinine (max)**	**Platelets (min)**	**Leukocyte count (max)**	**tPA**	**MPO**	**HNE**	**Histones**	**IL-8**
MPO	*r*	0.215	−0.582^**^	0.405^**^	0.571^*^	1.000	0.363^**^	0.329^*^	0.659^**^
	Sig.	0.123	0.000	0.003	0.021		0.008	0.016	0.000
	*n*	53	52	52	16	53	53	53	36
HNE	*r*	0.364^**^	−0.420^**^	0.296^*^	0.636^**^	0.363^**^	1.000	0.130	0.474^**^
	Sig.	0.007	0.002	0.033	0.008	0.008		0.352	0.004
	*n*	53	52	52	16	53	53	53	36
Histones	*r*	0.228^*^	−0.260	0.273	−0.101	0.329^*^	0.130	1.000	0.464^**^
	Sig.	0.036	0.063	0.050	0.632	0.016	0.352		0.004
	*n*	53	52	52	16	53	53	53	36
IL-8	*r*	0.138	−0.379^*^	0.292	0.536	0.659^**^	0.474^**^	0.464^**^	1.000
	Sig.	0.423	0.025	0.089	0.089	0.000	0.004	0.004	
	*n*	36	35	35	11	36	36	36	36

*The clinical variables creatinine, platelet and leukocyte counts are maximum or minimum values from the course of hospital stay whereas tPA and markers of neutrophil activation are from 1st day of hospitalization. r, Spearman's rho correlation coefficient; Sig., Significance of correlation (p); n, number of patients; MPO, Myeloperoxidase; HNE, Neutrophil elastase; IL-8, Interleukin-8; tPA, Tissue plasminogen activator*;

* *p < 0.05*,

** *p < 0.01*.

### Localization of MPO, HNE, and IL-8 in the kidneys of acute PUUV-HFRS

**Figure 2:**
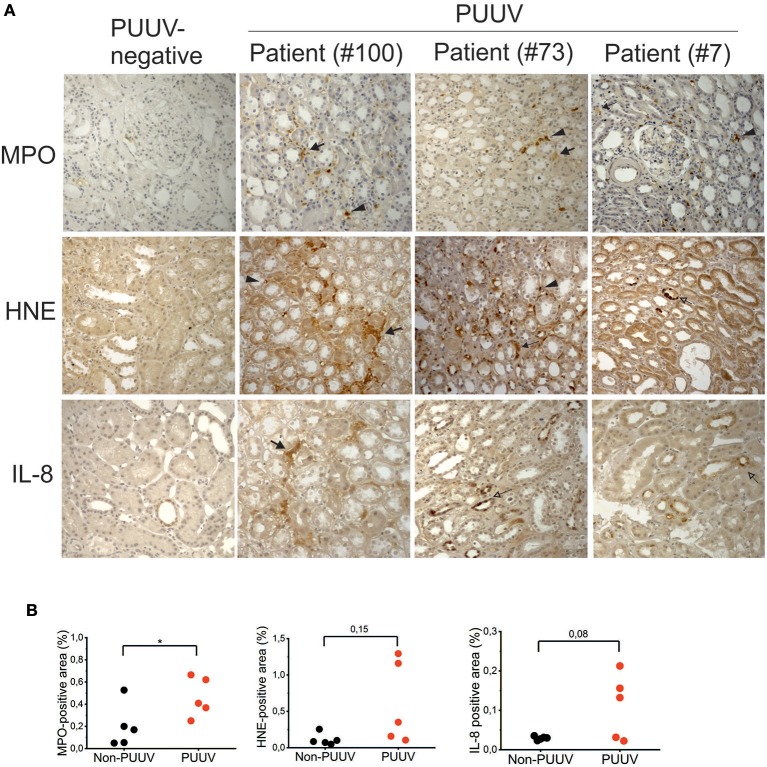
Immunohistochemical analysis of MPO, HNE and IL-8 in kidney sections from patients with acute PUUV-HFRS. Kidney sections from acute PUUV-HFRS or unrelated kidney diseases (*n* = 5 for both) were analyzed for the presence of MPO, HNE or IL-8 by standard immunohistochemical techniques and visualized by DAB staining. **(A)** Images from three cases with acute PUUV-HFRS and one PUUV-negative control section is shown. **(B)** The tissue area positive for MPO, HNE or IL-8 was evaluated as percentages in all sections and mean values ± standard deviation reported for acute PUUV-HFRS and PUUV-negative sections. Significant differences were assessed by student's *T*-test and statistically significant differences indicated as ^*^p < 0.05.

In order to investigate whether markers of neutrophil activation could also be detected in tissues of patients suffering from acute PUUV-HFRS, we made use of archival biopsies to detect the expression of MPO, HNE and IL-8 in patient kidneys by immunohistochemistry. We did not include histones in this analysis since we expected that differentiating NET-associated histones from viable cells would be challenging. Elevated expression of MPO, HNE and IL-8 could be detected more readily in acute PUUV-HFRS patients than PUUV-negative control patient samples (Figure 2A). By counting the tissue area staining positive for MPO, HNE, or IL-8 we could observe a statistically significant difference in MPO expression between PUUV positive and negative cases (*n* = 5; *p* = 0.04; Figure 2B). In the case of HNE and IL-8 higher expression was found in 2 and 3 PUUV cases, respectively, as compared to controls but this was not enough to reach statistical significance (*n* = 5; *p* = 0.08 and *p* = 0.15, respectively). The most prominent localization of MPO, HNE and IL-8 in HFRS patients was the tubulointerstitial space, in line with the diagnosis of tubulointerstitial nephritis. Therein we could observe both cell-associated (arrowheads) and extracellular expression (filled arrows) of MPO and HNE suggesting both neutrophil infiltration and activation, respectively. Interestingly, HNE and IL-8 localized also to in the tubular epithelial cells in two HFRS patients (empty arrows). IL-8 could also occasionally be observed in the tubular cells of PUUV-negative patients albeit with a lower intensity and frequency as compared to HFRS patients (Figures 2A,B). Taken together, these results show that MPO is a component of the inflammatory response toward PUUV in the kidneys of acute HFRS, possibly accompanied by HNE and IL-8, and suggest that neutrophils (and their activation products) infiltrate kidneys through the capillary endothelium. In addition, the current findings together with our previous observations of elevated IL-8 levels in urine of acute PUUV-HFRS (28) suggest that IL-8 is produced locally by kidney epithelial cells in PUUV-HFRS and likely acts as a chemotactic factor inviting neutrophil recruitment and extravasation in the kidney.

### Live PUUV does not activate neutrophils *in vitro*

**Figure 3:**
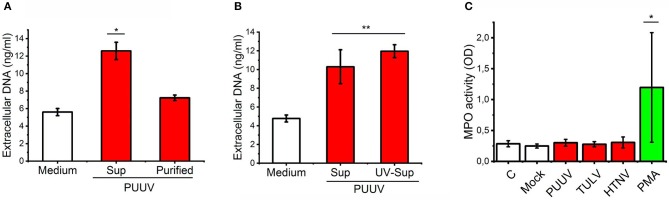
Live PUUV does not induce neutrophil activation. **(A)** Mock- or PUUV-containing Vero E6 supernatants (Sup) or purified PUUV were incubated with freshly isolated PMNs for 3 h (MOI 1) (*n* = 2). The release of extracellular DNA was assessed by an impermeable DNA binding fluorescent dye. **(B)** Mock-, live PUUV- or UV-inactivated PUUV-containing Vero E6 supernatants were incubated with PMNs and assessed for DNA release as in **(A)** (*n* = 3). **(C)** Purified PUUV, TULV or HTNV were incubated with fresh PMNs for 3 h (MOI 1) and peroxidase activity assessed from cell culture supernatants by TMB (*n* = 3). PMA-treated PMNs were used as a positive controls. Difference between groups were assessed by one-way ANOVA + Dunnett's multiple comparisons test and statistically significant differences indicated as either ^*^p < 0.05 or ^**^p < 0.01.

It has been shown that HTNV can directly bind CD18 integrin on neutrophils and induce NETosis (21). We wanted to determine whether PUUV can also induce NETosis which could explain our findings of neutrophil activation in acute PUUV-HFRS. We did this by incubating mock- or PUUV-infected Vero E6 cell culture supernatants or purified PUUV (with the same infectious titer) with freshly isolated PMNs and subsequently quantified the release of extracellular DNA from PMNs. NETosis was clearly induced above mock control by PUUV-containing Vero E6 supernatant but not with purified PUUV (Figure 3A). To elaborate on the finding that live PUUV does not cause NETosis, UV-inactivation of PUUV-infected Vero E6 supernatant did not affect its ability to induce NETosis (Figure 3B). These findings indicate that live PUUV alone is not capable of inducing NETosis but instead involves other factor/s released from hantavirus-infected Vero E6 cells supernatants which might act alone or synergistically with the inactivated virus. In order to analyze whether purified hantaviruses could induce PMN degranulation we incubated fresh PMNs with purified PUUV, TULV, HTNV, or PMA (as a positive control) and analyzed peroxidase activity in cell supernatants (Figure 3B). MPO activity was induced by PMA but not with any of the hantaviruses tested, suggesting that hantavirus do not induce significant PMN degranulation. These results imply that neutrophil activation in HFRS is not caused by direct contact with hantavirus but instead requires additional factors. Thus, we wanted to analyze whether PUUV-infected endothelial cells could play a role in neutrophil activation during HFRS.

### PUUV induces a pro-inflammatory phenotype in BECs

**Figure 4:**
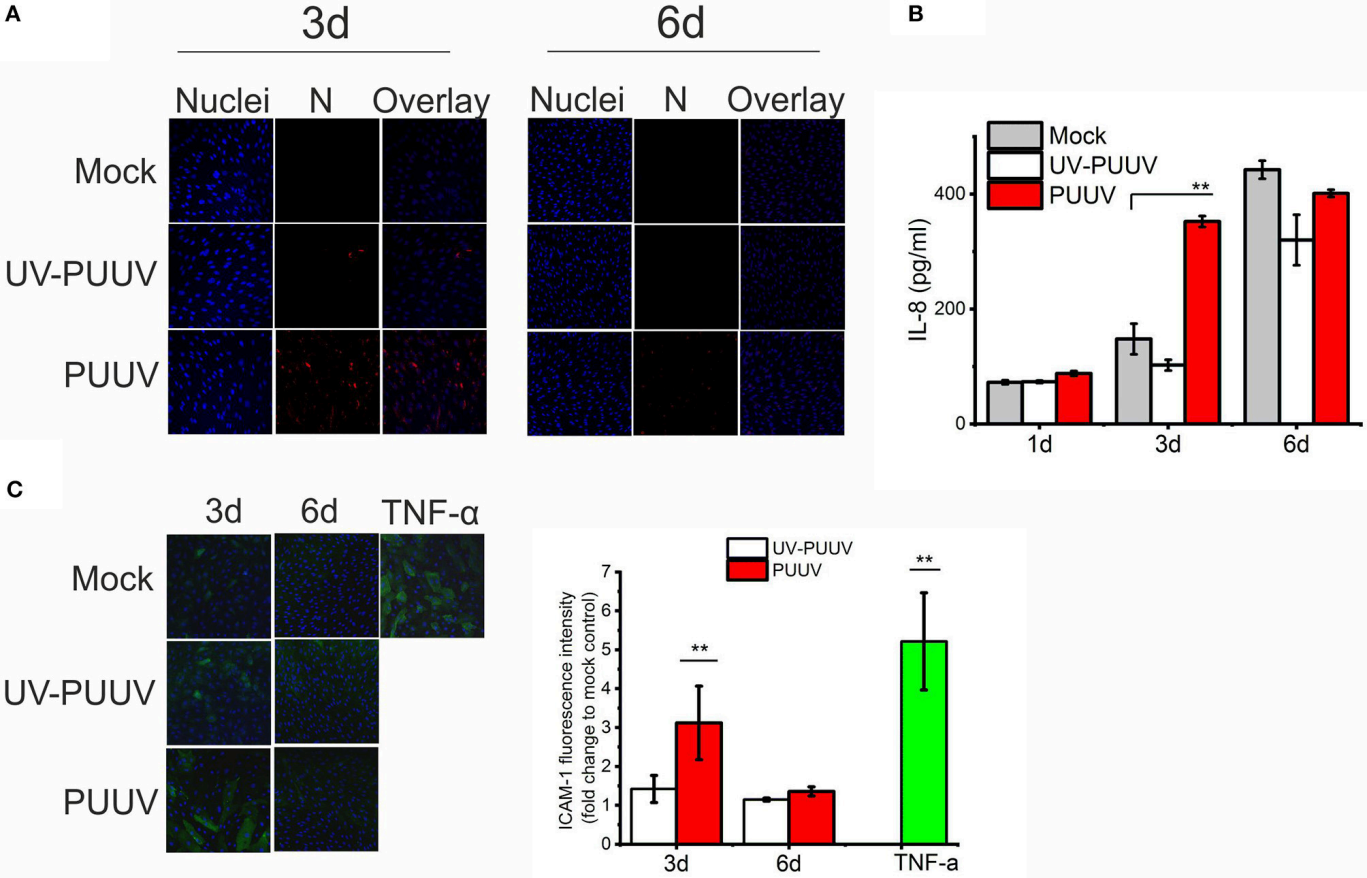
PUUV infection causes pro-inflammatory responses in BECs. **(A)** BECs were either mock-infected or infected with live or UV-inactivated PUUV (UV-PUUV) and assessed for viral nucleocapsid protein expression at 3 and 6 days post infection by immunofluorescence (red). The nuclei of BECs were visualized with Hoechst 33420 (blue). **(B)** IL-8 was measured from the respective supernatants of mock, UV-PUUV or PUUV-infected BECs by ELISA. **(C)** ICAM-1 expression in TNF-α treated or mock-, UV-PUUV or PUUV-infected BECs was visualized by immunofluorescence (green) and shown as an overlay with Hoechst 33258 staining (blue). Fluorescence intensity of ICAM-1 expression on BECs was quantified and reported as fold change to mock-infected cells. Differences between groups were assessed by one-way ANOVA with Dunnett's multiple comparisons test and statistically significant differences indicated as ^**^p < 0.01. *n* = 2 in all panels. Results shown are representatives of three independent experiments.

Endothelial cells are the prime target of hantavirus infection *in vivo* (2, 3). Thus, we hypothesized that PUUV could mediate neutrophil activation through infected endothelial cells. To test this, we either mock-infected or infected primary blood endothelial cells (BECs) with purified live PUUV or UV-inactivated PUUV (UV-PUUV). By visualizing the expression of viral nucleocapsid protein N in the cells by immunofluorescence we observed that BECs were close to 100% infected at 3 days post infection (dpi), followed by significant drop in infectivity levels at 6 dpi (Figure 4A). The downregulation of PUUV infection in BECs is type I interferon-mediated, as reported previously (29), and is probably due to MxA protein sequestering the viral N protein (30) We analyzed the supernatants of infected BECs for the presence of IL-8 by ELISA and observed that BECs infected with live PUUV upregulate the secretion of IL-8 at 3 dpi as compared to mock- or UV-PUUV infections (Figure 4B). However, longer culturing of mock- or UV-PUUV infected BECs also upregulated IL-8 in the cell supernatants which finally led to comparable, high levels of IL-8 irrespective of PUUV infection at 6 dpi. Since IL-8 could act as a general marker of inflammation (10), we wanted to see whether PUUV-infected BECs would upregulate also other inflammatory factors potentially important for neutrophil recruitment. Thus we assessed the expression of ICAM-1, a ligand for neutrophil-expressed CD11b/CD18 integrin complex, on the plasma membrane of BECs by immunofluorescence-based imaging and quantification assays. We found elevated levels ICAM-1 on the surface of PUUV-infected BECs as compared to mock- or UV-PUUV infected BECs at 3 dpi (Figure 4C). The elevated ICAM-1 levels remained only slightly lower as compared to TNF-α treated BECs, used as a positive control. At 6 dpi, ICAM-1 expression returned to baseline levels in PUUV-infected BECs concomitantly with the reduction of viral replication. As expected, TNF-α was found to be a robust inducer of ICAM-1 in BECs and thus we hypothesized that TNF-α could induce ICAM-1 also in PUUV-infected BECs. However, we did not find any evidence for TNF-α in PUUV-infected BEC supernatants by ELISA suggesting that the induction of ICAM-1 in PUUV-infected BECs is independent of this cytokine (data not shown).

### PMNs adhere to PUUV-infected BECs

**Figure 5:**
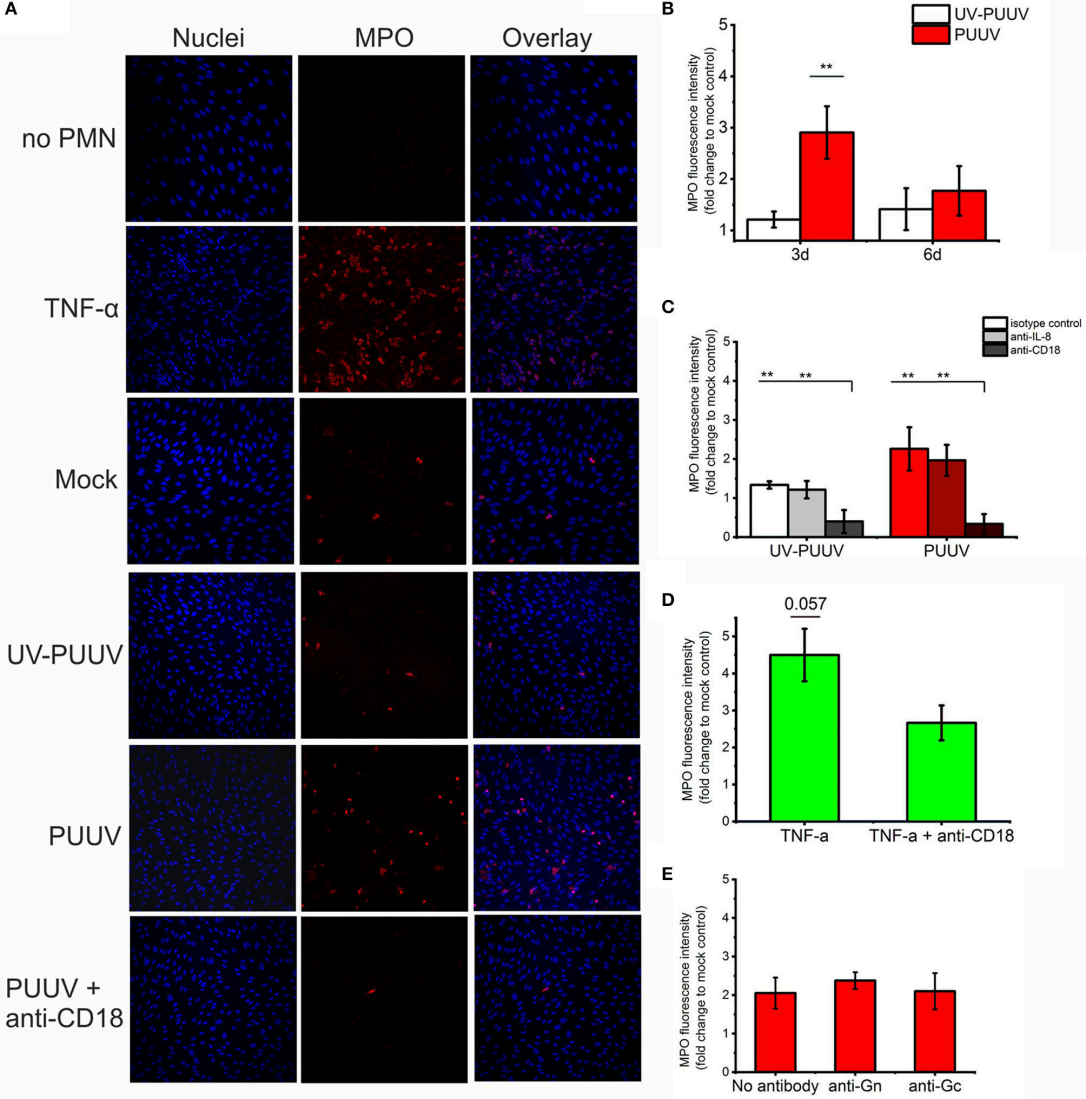
Binding of quiescent PMNs to PUUV-infected BECs. Freshly isolated PMNs were allowed to bind to mock-, UV-PUUV or live PUUV-infected BECs for 1 h at 3 or 6 days post infection (dpi). **(A)** PMNs bound to either TNF-α treated, UV-PUUV or PUUV-infected BECs were visualized at 3 dpi by immunofluorescence using MPO-specific antibody. The nuclei were stained with Hoechst 33420. **(B)** BEC-bound PMNs were quantified at 3 and 6 dpi as fold change to mock-infected cells. **(C)** PMNs were allowed to bind to PUUV-infected BECs at 3 days post infection in the presence of neutralizing antibodies to IL-8, CD18 or an isotype control and quantified as above. **(D)** PMNs were allowed to bind to BECs treated with TNF-α for 3 days in the presence or absence of CD18 antibody and quantified as above. **(E)** PMNs were allowed to bind at 3 days post infection to PUUV-infected BECs treated with Gn- or Gc-specific neutralizing antibodies and quantified as above. Statistically significant differences between groups were assessed either by student's *T*-test **(B,D)** or one-way ANOVA with Dunnett's multiple comparison test **(C,E)** and indicated as ^**^p < 0.01. *n* = 2 in all panels. Results shown are representatives of three independent experiments.

Next, we wanted to determine whether freshly isolated PMNs (containing mainly neutrophils) could adhere to PUUV-infected BECs which express elevated levels of IL-8 and ICAM-1. Fresh PMNs were incubated with mock-, UV-PUUV or live PUUV-infected BECs for 1 h, non-bound PMNs removed by washing and remaining numbers of BEC-bound PMNs determined by immunofluorescence-based imaging and quantitation of MPO (used as a marker of PMNs). In addition to MPO, bound PMNs were differentiated from BECs based on their segmented nuclear morphology by DNA staining. We found that PMN binding to PUUV-infected BECs was significantly elevated as compared to mock- or UV-PUUV infected BECs at 3 dpi, but not at 6 dpi (Figures 5A,B), consistent with the elevated expression of ICAM-1 and higher virus replication in PUUV-infected BECs at 3 dpi. As positive and negative controls, we used TNF-α treated BECs and PUUV infected-BECs without PMN incubation, respectively. Based on these results we consistently allowed PMNs to bind the 3-day BEC cultures for 1 h in the following experiments assessing PMN-BEC interaction as shown in Figure 5.

We wanted to analyze further whether the binding of PMNs to PUUV-infected BECs is mediated by chemotactic signals driven by IL-8 and/or is dependent of the neutrophil-expressed CD11b/CD18 binding to ICAM-1 on BECs. We did this by incubating PMNs with either BECs infected with either live PUUV or UV-PUUV as the negative control for infection in the presence of neutralizing antibodies to IL-8 or CD18. We could determine that binding to PUUV-infected BECs was dependent on CD11b/CD18 on neutrophils but not on the presence of IL-8 (Figures 5A,C). Not surprisingly, CD11b/CD18 seemed to mediate the adhesion of PMNs also to TNF-α treated BECs, although less dramatically (Figure 5D). The fact that CD18 neutralizing antibody was not as efficient in blocking PMN adhesion to TNF-α treated BECs could be due to the excess level of ICAM-1 upregulated by TNF-α as compared to PUUV infection (Figure 4C). Given that HTNV has been shown to bind CD18 integrin (21) we also tested the effect of viral glycoprotein (Gn and Gc) neutralizing antibodies (31) on PMN-BEC interaction. However, we could not observe any significant effects (Figure 5E) by Gn or Gc neutralizing antibodies suggesting that PMN adhesion to infected BECs is mediated by host-derived inflammatory factors but not viral proteins. Unfortunately, we could not reliably determine the role of ICAM-1 in PMN-BEC interaction since the presence of ICAM-1 specific antibodies resulted in further elevated binding of PMNs to BECs regardless of BECs being infected with PUUV or not (data not shown). We hypothesize that this phenomenon could be due to antibody-mediated cross-linking of neutrophil- and BEC-associated ICAM-1 proteins. In addition, ICAM-1 antibodies are known to activate cross-linking and activation of ECs on cell surfaces in some conditions (32) which could potentially explain this finding.

### PUUV-infected BECs induce PMN degranulation

**Figure 6:**
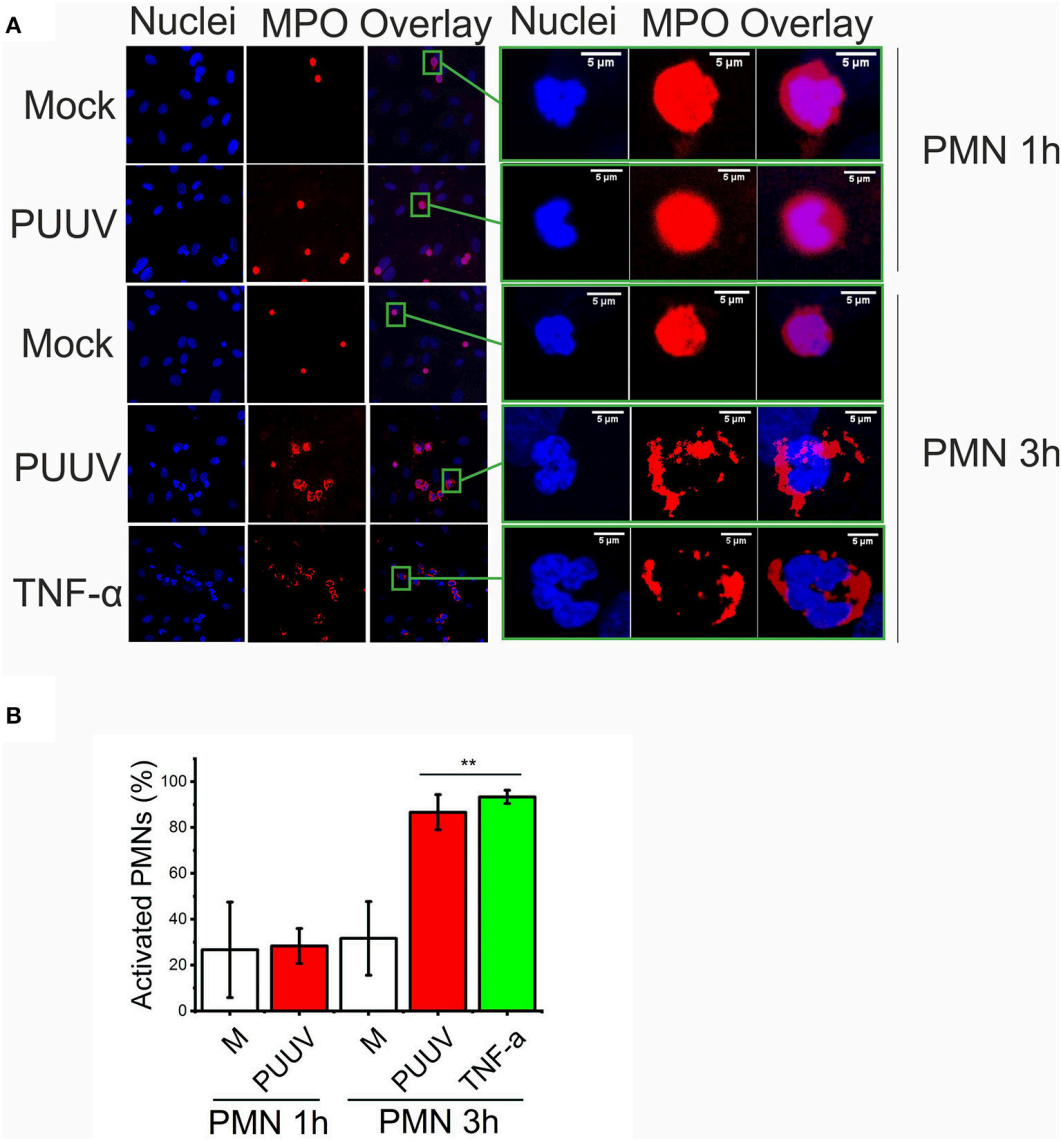
PUUV-infected BECs induce PMN degranulation. **(A)** PMNs were allowed to bind to mock- or PUUV-infected BECs at 3 days post infection for 1 or 3 h. BECs treated with TNF-α for 1 day and exposed to PMNs for 3 h were used positive control for PMN activation. PMN activation was assessed by morphological changes of bound PMNs as observed by immunofluorescence staining for MPO (red) and nuclei (Hoechst 33420, blue). On the right, the fluorescence image of one individual BEC-bound PMN from each treatment group is enlarged for better visualization of PMN activation. **(B)** The percentage of swollen nuclei with concomitant changes in MPO expression from diffuse cytoplasmic to plasma membrane localization were counted in individual wells and mean values ± standard deviation reported. Statistically significant differences between groups is assessed by student's *T*-test and indicated as ^**^p < 0.01. *n* = 3. Results shown are representatives of two independent experiments.

Next, we wanted to determine whether more extensive co-culturing of PMNs and PUUV-infected BECs could result in PMN activation (degranulation or NETosis) which could explain our findings of increased levels of extracellular neutrophil markers in PUUV-HFRS patients (Figures 1, 2). We observed a time-dependent morphological change in PMNs bound to PUUV-infected BECs but not in mock- or UV-PUUV infected BECs which was visible after 3 but not 1 h of co-culture (Figures 6A,B). This was evident by increased nuclear swelling of PMNs together with altered localization of MPO from diffuse to plasma membrane-associated staining. Furthermore, MPO could be observed also extracellularly, which strongly suggests that PMN degranulation was taking place. The fluorescent phenotype of PMNs bound to PUUV-infected BECs was strikingly similar although less pronounced as observed for PMNs bound to TNF-α treated cells after a 3 h of co-culture. We did not find any evidence for NETosis in PMNs bound either on PUUV-infected or TNF-α treated cells or by PUUV-infected BEC supernatants (data not shown) in these experimental conditions.

### Antiviral effect of PMNs is mediated by protease release *in vitro*

**Figure 7:**
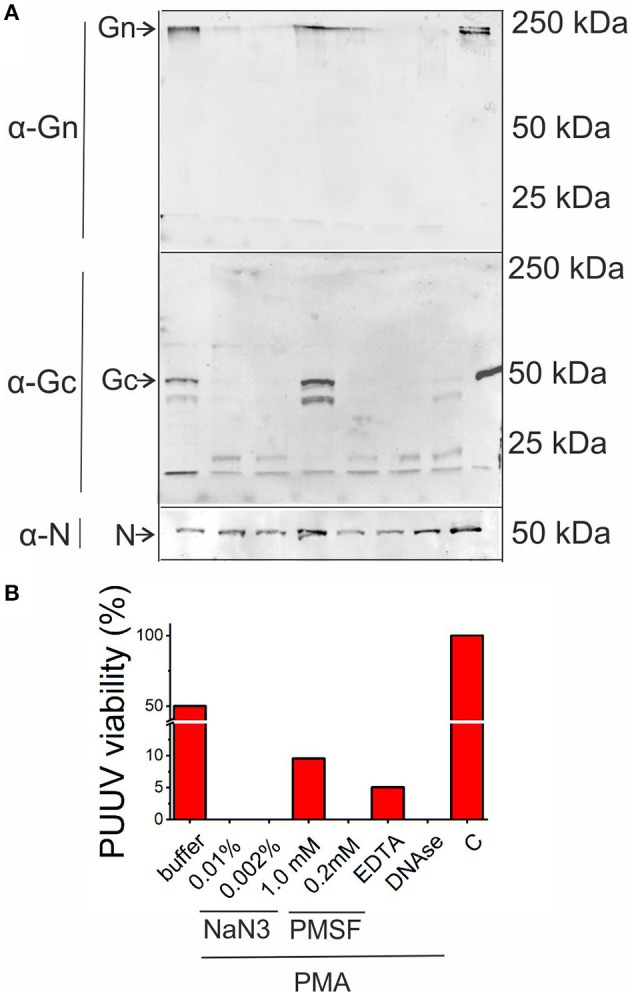
Antiviral activity of activated PMNs. Purified PUUV was incubated with quiescent or PMA-activated freshly isolated PMNs in the presence of inhibitors for MPO (0.01 or 0.002% NaN_3_), serine proteases (1 or 0.2 mM PMSF), metalloproteinases (1 mM EDTA) or NETs (10 U/ml DNAse) and **(A)** subjected to immunoblotting for viral proteins Gn, Gc and N protein and infectious titer measurement in Vero E6 cells **(B)**. C, Control without the addition of PMNs.

Finally, we wanted to determine whether neutrophils possess antiviral function and if so, by which mechanism. We incubated purified PUUV with freshly isolated PMNs, which were either non-activated or activated with PMA. Furthermore, incubations of virus with PMA-activated PMNs were performed in the presence of one of the following inhibitors: NaN_3_ (an inhibitor of MPO activity), phenylmethylsulfonylfluoride (PMSF; inhibitor of serine proteases), EDTA (inhibitor of metalloproteinases) and DNAse (degradation of NETs). After incubation, samples were subjected to immunoblotting in order to detect degradation of viral structural proteins Gn, Gc and N protein (Figure 7A) and virus titer determinations in Vero E6 cells (Figure 7B). The PUUV Gc and N proteins migrated as expected based on their molecular size of ~54 and 50 kDa, respectively, whereas Gn is known to form SDS-stabile tetramers of ~240 kDa in SDS-PAGE [Figure 7A, (27)]. PMA-treated PMNs complete destroyed PUUV infectivity and the surface glycoproteins Gn and Gc while it has almost no effect on N protein, which is retained inside the virus particle, whereas quiescent PMNs decreased PUUV viability only marginally (50%) with the concomitant low-level cleavage of Gc. PMSF totally blocked the cleavage of Gn and Gc while retaining almost 10% of PUUV infectivity indicating strong involvement of serine proteases (such as HNE) in the antiviral effect of activated PMNs. Also, EDTA treatment retained significant levels of infectivity (5%) although not being able to block the degradation of Gn and Gc significantly. However, NaN_3_ or DNAse treatments had no effect on the ability of PMA-treated PMNs to kill virus or degrade its glycoproteins, indicating that MPO or the formation of NETs do not play a direct role in the antiviral effects of PMNs in this experimental setup. The fact that the infectivity of PUUV was not significantly reduced after treatment with quiescent PMNs is further proof (see Figure 3) that hantaviruses are unable to significantly activate PMNs.

## Discussion

We observed elevated levels of circulating extracellular MPO, HNE and histones in the acute stage of PUUV-caused HFRS as compared to the recovery stage and healthy controls. MPO and HNE are expressed in neutrophil granules and released to the extracellular space through neutrophil degranulation or alternatively as components of NETs. Histones, on the other hand, are essential components of NETs but could be also released from other types of dying cells. The circulating levels of histone H3 correlated with MPO and HNE suggesting that all these factors are released simultaneously from neutrophils during NETosis, which presence in acute PUUV-HFRS has been reported previously (21). Interestingly, we found strong positive correlations between the levels of HNE or histone H3 and variable reflecting the severity of AKI (maximum creatinine); indicating that neutrophils could play a role in mediating kidney damage, the hallmark of HFRS. We could observe elevated expression of MPO in kidneys of acute PUUV-HFRS where it mainly localized to the interstitial space surrounding tubules, supporting earlier findings that infiltration of neutrophils into kidneys is a part of the inflammatory response toward PUUV (7).

In addition, we found that the major chemotactic factor for neutrophils, IL-8, is upregulated in the acute stage of PUUV-HFRS in blood, as previously reported (33), and correlated strongly with neutrophil activation markers (MPO, HNE and histone H3). This was also accompanied by a tendency of higher IL-8 expression also in the kidneys of acute PUUV-HFRS patients. The kidney IL-8 was found to be localized to the tubulointerstitial space, similarly to MPO and HNE, but also to tubular epithelial cells. This suggests IL-8 could be expressed directly by the kidney in PUUV-HFRS and plays a major role in recruiting neutrophils into this tissue. Furthermore, local expression of IL-8 in the kidney probably explains the high levels of IL-8 found in the urine of PUUV-HFRS patients (28).

AKI is the hallmark symptom of HFRS but the disease manifests as systemic vascular dysfunction as indicated by capillary leakage in several organs (1). It is of interest to note that neutrophil activation markers not only correlate with kidney injury, but also with different hematological parameters indicating the extent of thrombocytopenia, leukocytosis and fibrinolysis (low platelets, high leukocytes and high tPA, respectively). This suggests that neutrophil activation either contributes to or is in fact caused by the same underlying factors which lead to capillary leakage in PUUV-HFRS and is not an isolated phenomenon which effect would be restricted to the kidney. This calls for future studies aimed at determining the extent of neutrophil activation also in other forms of hantavirus-mediated diseases such as HCPS which severely affect lungs instead of kidneys. In fact, neutrophils have been shown as the main culprits of pulmonary vascular permeability in mouse model of HTNV infection (25).

A previous report indicates that HTNV binds CD11b/CD18 integrin complex on the surface of neutrophils and, as shown using HTNV-containing Vero E6 cell culture supernatants, activates NETosis in freshly isolated PMNs (21). Given the high degree of similarity between different hantaviruses (1), we hypothesized that also PUUV is be able to activate in NET formation in PMNs. However, we observed that, while PUUV-containing Vero E6 cell culture supernatant was able to activate NETosis, purified PUUV was not (Figure 3A), suggesting that NETosis is not mediated by live, intact virus but rather other factor/s present in the cell culture supernatant of infected Vero E6 cells. We therefore sought for an alternative mechanism of neutrophil activation in PUUV-HFRS and turned our attention toward microvascular endothelial cells, the prime target of hantavirus infection *in vivo*. To begin with, we found that PUUV-infected blood microvascular cells (BECs) secreted elevated levels of IL-8 and expressed more ICAM-1 on their surface than non-infected BECs; suggesting that PUUV infection induces a pro-inflammatory phenotype in BECs. Importantly, elevated expression of ICAM-1 has been also detected in kidneys of acute PUUV-HFRS (8). We observed that freshly isolated PMNs adhered to PUUV-infected, pro-inflammatory BECs which was dependent on CD11b/CD18 integrin complex on the surface of neutrophils but not IL-8 or viral glycoproteins. We then asked whether the BEC-adhered PMNs would become activated to explain the observed elevated levels of MPO, HNE and histones in patients. We found that extensive co-culturing of fresh PMNs together with PUUV-infected BECs resulted in morphological changes in PMNs, which suggested degranulation of PMNs. However, we did not find any evidence of increased NETosis in these co-cultures.

These results imply that PUUV infection causes pro-inflammatory changes in endothelial cells to attract and activate neutrophils on the surface of the endothelium by an IL-8 dependent mechanism. It is previously noted that pro-inflammatory endothelial cells can induce NETosis partially through EC-derived IL-8 causing endothelial cell death (34). The interactions of neutrophils with the endothelium in inflammatory conditions is well-documented (9, 12, 35, 36) and typically these events are not associated with clinically significant vascular permeability. In typical inflammatory reactions, however, the chemotactic signals that induce inflammation originate from tissues from where neutrophil diapedesis across the endothelium is locally dictated. This is in contrast to hantavirus infections where the viruses replicate in endothelial cells to cause systemic inflammation of the vasculature. In addition to endothelial cells, hantavirus glycoproteins have been detected in renal tubular cells in acute HTNV-caused HFRS (37), where we observed increased levels of HNE and IL-8 in this study, suggesting that hantavirus-infected tubular cells could behave similarly to infected endothelial cells and recruit and activate neutrophils by an IL-8 dependent mechanism. The fact that depletion of neutrophils suppress lung pathology in a mouse model for HTNV certainly supports the idea of neutrophils as the driving force of HFRS pathogenesis (25). Similar mechanisms could play a role also in other hemorrhagic fevers (38). It is known that for instance that flavi- and filoviruses induce pro-inflammatory changes in endothelial cells (39–41). Of note, ICAM-1 polymorphism is associated with dengue disease severity (42).

Neutrophils are well-known for their antibacterial effects but their ability to counteract viral infections are only beginning to be unraveled (17, 35, 43). Early research indicates that defensins, which are peptides released from neutrophils upon activation, show antiviral activity against enveloped viruses (44). MPO can also cause oxidative damage to viruses upon neutrophil oxidative burst and degranulation (45). Lately, the antiviral effects of NETs have been implicated (46). In the present study we observed that serine proteases (such as HNE) play a major role in the virucidal function of neutrophils against PUUV. However, we did not obtain evidence for a direct role of NETs or MPO-mediated oxidative effects in killing PUUV. Furthermore, the fact that PUUV was not killed by PMNs which were non-activated at the start of the experiment, show that PUUV doesn't directly cause significant protease release by PMNs. The inability of PMNs to produce NETs through direct contact with hantaviruses is not surprising since typically NETs are produced in response to larger microbes (47) but given the known expression of several Toll-like receptors by neutrophils (48), their inability to release proteases (degranulate) in response to PUUV is peculiar. However, some viruses are known to block the activation of neutrophils (49, 50) and whether this is also the case for hantaviruses remain a topic for future studies.

To conclude, we have found that patients suffering from acute PUUV-caused HFRS show elevated levels of circulating MPO, HNE, histone H3 and IL-8 indicating neutrophil activation through degranulation and/or NETosis. The expression levels of these markers positively correlate with parameters reflecting disease severity suggesting that neutrophil activation could play a major role in the pathogenesis of HFRS. Mechanistically, our data indicates that neutrophil activation is more likely to occur indirectly via virus-infected microvascular endothelial cells rather than directly through virus contact with neutrophils. Future studies are still needed to elucidate whether either neutrophil degranulation or NETosis has the stronger impact on hantavirus pathogenesis.

## Author contributions

TS and AV conceived and designed the research. TS performed the experiments and analyzed the data. SM and JM contributed to sample collection. TS wrote the paper. All authors reviewed the manuscript.

### Conflict of interest statement

The authors declare that the research was conducted in the absence of any commercial or financial relationships that could be construed as a potential conflict of interest.
